# Disentangling Cation and Anion Dynamics in Li_3_PS_4_ Solid Electrolytes

**DOI:** 10.1021/acs.chemmater.2c02637

**Published:** 2022-11-09

**Authors:** Frazer N. Forrester, James A. Quirk, Theodosios Famprikis, James A. Dawson

**Affiliations:** † Chemistry − School of Natural and Environmental Sciences, Newcastle University, Newcastle upon TyneNE1 7RU, U.K.; ‡ Department of Radiation Science and Technology, Faculty of Applied Sciences, Delft University of Technology, 2629JBDelft, The Netherlands; § Centre for Energy, Newcastle University, Newcastle upon TyneNE1 7RU, U.K.

## Abstract

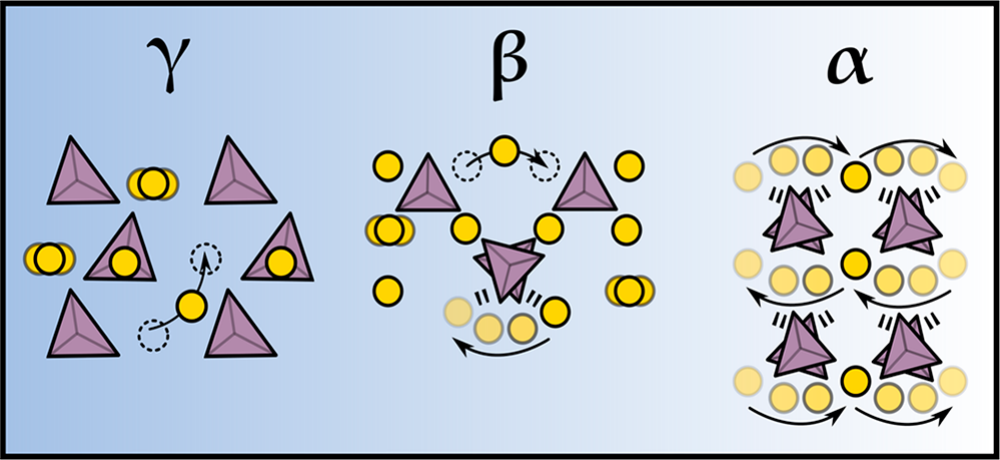

A prerequisite for the realization of solid-state batteries is the development of highly conductive solid electrolytes. Li_3_PS_4_ is the archetypal member of the highly promising thiophosphate family of Li-ion conductors. Despite a multitude of investigations into this material, the underlying atomic-scale features governing the roles of and the relationships between cation and anion dynamics, in its various temperature-dependent polymorphs, are yet to be fully resolved. On this basis, we provide a comprehensive molecular dynamics study to probe the fundamental mechanisms underpinning fast Li-ion diffusion in this important solid electrolyte material. We first determine the Li-ion diffusion coefficients and corresponding activation energies in the temperature-dependent γ, β, and α polymorphs of Li_3_PS_4_ and relate them to the structural and chemical characteristics of each polymorph. The roles that both cation correlation and anion libration play in enhancing the Li-ion dynamics in Li_3_PS_4_ are then isolated and revealed. For γ- and β-Li_3_PS_4_, our simulations confirm that the interatomic Li–Li interaction is pivotal in determining (and restricting) their Li-ion diffusion. For α-Li_3_PS_4_, we quantify the significant role of Li–Li correlation and anion dynamics in dominating Li-ion transport in this polymorph for the first time. The fundamental understanding and analysis presented herein is expected to be highly applicable to other solid electrolytes where the interplay between cation and anion dynamics is crucial to enhancing ion transport.

## 1 Introduction

With the demand for electricity ever increasing and current energy supplies highly dependent on finite fossil fuel reserves, next-generation battery materials are taking center stage as critical drivers for both the electrification of transport and the storage of intermittently produced renewable power. Solid-state batteries (SSBs) are attracting increased attention as a result of their potentially significant improvements in energy density and safety when compared with their liquid-based electrolyte counterparts.^1−8^ However, although promising, this technology remains in relative infancy, with its success highly dependent on discovering and developing solid electrolyte (SE) materials with sufficient ionic conductivity (i.e., ∼10 mS cm^–1^ at room temperature).^1−8^

Many structural families have been widely considered for SEs, each with unique trade-offs for SSB applications.^4^ While early research primarily focused on conventional oxides, such as silicates^9^ and phosphates,^10^ their sulfide analogues are now recognized as uniquely promising, subject to their inherently high room-temperature ionic conductivity and excellent formability. Ionic conduction is known to be largely dependent on the radius and polarizability of the mobile ions, with the replacement of O with S weakening the interactions between Li ions in the sublattice, thereby increasing their mobility.^11^ Commensurately, investigations by Wang et al. proposed body-centered-cubic (BCC) packing of the anion (sulfur) sublattice to be particularly conducive to high Li-ion diffusivity,^12^ as observed in fast Li-ion conducting sulfide materials such as Li_10_GeP_2_S_12_ and Li_7_P_3_S_11_.^12,13^ Other notable characteristics of sulfide SEs, such as their low electronic conductivities and high lithium transference numbers (≈1), are also conducive to their utilization in SSBs.^2,13−18^

Among sulfide electrolytes, Li_3_PS_4_ is considered the archetypal member of the very promising thiophosphate family. Its potential stability with lithium metal and high ionic conductivity in nanoporous form^19^ have made it the subject of or prelude to many experimental and computational studies.^4−7,20−23^ Li_3_PS_4_ primarily exists as three main polymorphs: γ, β, and α, each with varying structural and physical properties. The γ polymorph (*Pmn*2_1_) exhibits low room-temperature ionic conductivity (∼3 × 10^–7^ S cm^–1^).^24−26^ At higher temperatures (*T* > 500 K), the γ polymorph transforms into the metastable β polymorph (*Pnma*),^24−29^ followed by conversion to the α polymorph (*Cmcm*) at *T* > 740 K, which is predicted to be a superionic conductor.^25,30^ The stabilization of the nanoporous β polymorph at room temperature (via solution synthesis in tetrahydrofuran) results in a high Li-ion conductivity of the order of 10^–4^ S cm^–1^,^19^ with other solution-based syntheses giving similarly promising results.^31−35^

It is understood that the significant differences in these polymorphs with regards to ionic conductivity are the result of transformations in the orientation of PS_4_ tetrahedra. These polymorphic alterations see the hexagonal close-packed (HCP)-like close packing arrangement in the γ polymorph become successively distorted through β to α, approaching the more favorable BCC-like framework with regards to conduction.^30^ A fourth δ polymorph (*P*42_1_*c*) has also been identified, although it is only obtainable under high pressure and thus transcends current practical operation.^36^ While the structures of these various temperature-dependent polymorphs have been relatively well characterized, the underlying atomic-scale features that govern the relationship between cation and anion dynamics remain unclear. Previous work on these materials has been largely devoted to the relevance of static structural manipulation, with significant attention given to the effects, rather than the origin, of subsequent correlative events inherent to fast-ion conduction.

“Correlation” in the broadest sense is the foundation of dynamics in the solid state, with microscopic events correlating to macroscopic properties. However, the term has become somewhat ambiguous in the literature, and while its attribution to fast-ion conduction is undoubtable, it is important that we consider the individual mechanisms it encapsulates distinctly.

Correlation exists between the successive jumps of ions and the vacancies they create. In a typical solid, this often results in diffusivities lower than anticipated, with a finite probability of a diffusing atom making a reverse jump (i.e., to the recently vacated site). Conversely, when considering fast ion conductors (e.g., Li_10_GeP_2_S_12_^37^), a high concentration of mobile carriers coupled with a low activation energy landscape sees the probability of “cooperative” (or “concerted”) motion become much more likely. This is where the simultaneous movement of multiple ions in tandem sees the creation of “string-like” migration pathways of which atoms can follow; this of course becomes more probable at elevated temperatures and may occur more readily at higher defect concentrations.^37−40^

Another category of correlation links the movement of nonmigrating atoms and their incidental promotion of the mobile species. In this context, several seminal studies have highlighted a process rudimentarily termed the “paddlewheel” (or “rotor” effect),^41−45^ relating the ionic mobility of one ion with the rotational dynamics of complex (polyanionic) counterions. It is proposed that the reorientation of these complexes opens lower energy migration pathways between lithium sites, thereby facilitating faster Li-ion motion. While these terms (i.e., rotation, “paddlewheel”, rotor, and so on) are largely accepted in the literature, they are often ill-defined and applied equally to describe both complete free rotations of tetrahedra and weak oscillatory rotational dynamics, i.e., libration.^41,43,44^

In contrast to recent contributions that have begun to elucidate the topological dependence and concomitant roles these correlative mechanisms have in enhancing Li-ion conductivity,^45^ here the magnitude and dominance of these effects are assessed and quantified for the three main polymorphs of Li_3_PS_4_ for the first time. We provide the first comprehensive study of all three polymorphs of Li_3_PS_4_ simultaneously using molecular dynamics (MD) to derive the fundamental mechanisms and structural morphologies that underpin fast-ion diffusion in this material. By considering the spatial, temporal, and energetic correlations that both mediate and restrict ion transport, we independently observe lithium migration via the concerted motion of lithium ions and the libration of the PS_4_ tetrahedra and elucidate the magnitude of their effects on the overall Li-ion transport for each polymorph of Li_3_PS_4_.

## 2 Methodology

The MD simulations presented herein are based on established techniques and have been successfully utilized to investigate ion transport in a vast array of Li-ion conductors.^46−50^ The high computational efficiency of classical MD simulations allows for the use of sufficiently large supercells and time scales that are orders of magnitude greater than those that can be achieved with *ab initio* MD (AIMD), thereby enabling the capture of statistically significant variation in diffusivities.

The interatomic potentials used in this study (see Table S1) were developed by Kim et al.^48^ based on a combination of structural (lattice parameters and positions) and thermomechanical (Young’s, shear, and bulk moduli and Poisson’s ratio) properties obtained from density functional theory (DFT) calculations and experiments. A cutoff of 12 Å was applied to all the potentials. This model has been successfully used to study Li-ion transport in both crystalline and glassy thiophosphate solid electrolytes.^48^ To demonstrate the efficacy of this potential model, the calculated lattice parameters and atomic positions of γ-, β-, and α-Li_3_PS_4_ were compared to values obtained from X-ray diffraction^25^ and are in good agreement (see Tables S2 and S3). The validity of the potential model was further confirmed by calculating the energies of the three polymorphs at 0 K. In agreement with the established temperature-dependent polymorph transitions, the order of relative stability for the three Li_3_PS_4_ polymorphs was γ (0 eV per formula unit (fu)) > β (0.12 eV per fu) > α (0.36 eV per fu). The crystal structures of γ-, β-, and α-Li_3_PS_4_ are presented in Figure S1.

MD simulations were performed using the Large-scale Atomic/Molecular Massively Parallel Simulator (LAMMPS) package^51^ for temperatures in the range of 400–1000 K at increments of 100 K. Statistical properties were then obtained from suitably long 10 ns simulations utilizing a 1 fs time step in the NVT ensemble with the Nosé–Hoover thermostat and an initial equilibration performed using the NPT ensemble for several nanoseconds. Test simulations using the NPT ensemble for the entire 10 ns were also performed, but its impact on the Li-ion diffusion was negligible (<5%), with no significant change in Li-ion dynamics. Supercells of ∼14000 ions were constructed for each polymorph by repetition of the perfect unit cells (9 × 10 × 10, 5 × 9 × 10, and 8 × 7 × 8 unit cells for γ-, β-, and α-Li_3_PS_4_, respectively). Given that Li-ion diffusion was not observed in γ-Li_3_PS_4_ at 400–1000 K due to its ordered structure, it was necessary to introduce Li vacancies into the structure to promote long-range Li-ion diffusion, and this was achieved by randomly removing 1% of the Li ions from the system to produce Li_2.97_PS_4_. The same process was repeated for β- and α-Li_3_PS_4_ to enable a valid comparison between the three polymorphs. Unless explicitly stated otherwise, the results presented are for the Li_2.97_PS_4_ systems. For each composition, three randomly generated structures were simulated, and results are presented as averages of the three. Charge compensation was achieved by smearing the residual charge over all remaining Li ions. Self-diffusion data for the Li ions were derived from the time-averaged mean-square displacement (tMSD) according to
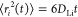
1where ⟨*r*_*i*_^2^(*t*)⟩ is the tMSD, *D*_Li_ is the diffusion coefficient for Li, and *t* is time. Example tMSD plots are presented in Figure S2.

## 3 Results and Discussion

### 3.1 Li-Ion Diffusion in Li_3_PS_4_

**Figure 1:**
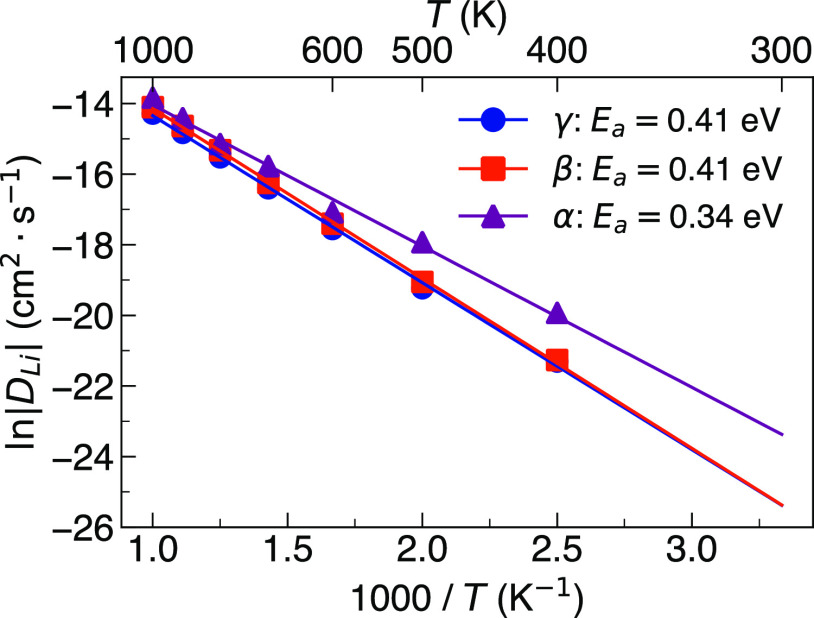
Arrhenius dependence of Li-ion diffusion in γ-, β-, and α-Li_3_PS_4_.

Li-ion diffusion in the three main polymorphs of Li_3_PS_4_ was first investigated at a range of temperatures (400–1000 K) to provide a reliable reference when considering the effect of subsequent dynamical properties. As indicated by the plots of Li-ion diffusion for the polymorphs with 1% of Li ions removed in Figure 1, the diffusion coefficients follow the expected Arrhenius relation in all three polymorphs. The equivalent Li-ion diffusion plots for the stoichiometric polymorphs are presented in Figure S3. Our simulations predict higher Li-ion diffusion in β-Li_3_PS_4_ compared to γ-Li_3_PS_4_ (for both the Li vacancy and stoichiometric systems) over the entire simulated temperature range, in agreement with the conductivities of these materials determined from electrochemical impedance spectroscopy.^19,25^ α-Li_3_PS_4_ exhibits the highest Li-ion diffusion of the three polymorphs. It is noteworthy that the introduction of a small concentration of Li vacancies to γ-Li_3_PS_4_ is sufficient to increase its level of Li-ion diffusion to close to that of β-Li_3_PS_4_ (see Figure 1).

The calculated Li-ion diffusion coefficients at 400 K for γ-, β-, and α-Li_3_PS_4_ from Figure 1 are 5.6 × 10^–10^, 5.6 × 10^–10^, and 2.28 × 10^–9^ cm^2^ s^–1^, respectively. Although these values are in good agreement with previous values obtained from classical MD and AIMD simulations of γ-Li_3_PS_4_ (∼3 × 10^–9^ cm^2^ s^–1^ at 450 K)^48^ and glassy Li_3_PS_4_ (∼2 × 10^–9^ cm^2^ s^–1^ at 400 K),^16^ respectively, they are over an order of magnitude lower than those obtained from nuclear magnetic resonance (NMR) experiments and AIMD simulations of 3 × 10^–8^ cm^2^ s^–1^ at 373 K^52^ and 1 × 10^–8^ cm^2^ s^–1^ at 383 K,^29^ respectively, for β-Li_3_PS_4_. We return to this discrepancy in the next section. We are not aware of any experimentally determined Li-ion diffusion coefficients for the low- and high-temperature γ and α polymorphs, respectively. It is noteworthy that we were unable to replicate the remarkably high Li-ion diffusion of ∼3 × 10^–6^ cm^2^ s^–1^ at 400 K for α-Li_3_PS_4_ predicted by AIMD.^30^ Real-space trajectories for the Li ions in the three polymorphs at 400 K are presented in Figure S4. Given that detailed experimental and computational analyses of the dominant Li-ion migration pathways in γ-, β-, and α-Li_3_PS_4_ have been discussed elsewhere,^30,45,57,61^ they are not repeated here.

The activation energies for Li-ion diffusion in the three polymorphs are also presented in Figure 1. An activation energy of 0.41 eV was obtained for both γ- and β-Li_3_PS_4_. A notably lower value of 0.34 eV was found for α-Li_3_PS_4_. Marginally higher values of 0.40 and 0.48 eV were obtained for stoichiometric β- and α-Li_3_PS_4_, respectively, as shown in Figure S3. Validation of these values is not straightforward given the broad distribution of results in the literature from both experimental and computational methods. The largest activation energies of the three polymorphs have generally been reported for γ-Li_3_PS_4_ at ∼0.50 eV from impedance spectroscopy^24,30^ and 0.70 eV from DFT simulations.^53^

For β-Li_3_PS_4_, the activation energies from impedance spectroscopy are lower and typically in the range of 0.30–0.50 eV.^19,31,54^ Activation energies of 0.40 eV for macroscopic diffusion and 0.09 eV for local jumps have been obtained for β-Li_3_PS_4_ from NMR.^55^ A similarly wide range of values have also been observed with both DFT and MD simulations.^27,28,53,56^ Nevertheless, it is noteworthy that very low activation energies of 0.22 and 0.16 eV have also been reported for γ- and β-Li_3_PS_4_, respectively, by Homma et al.^25^

Unfortunately, a full experimental characterization of the Li-ion transport properties of the high-temperature α polymorph is not yet available for comparison due to the difficulty associated with its stabilization at room temperature.^30,57^ AIMD simulations have, however, been completed, and a notably low Li-ion activation energy of 0.18 eV was found.^30^ This value is clearly far lower than that calculated here (i.e., 0.34 eV), but a reliable comparison of the two is limited by the fact that the previous AIMD study was conducted for a small system size and short MD runs of 50 ps. It is known that for systems with highly correlated diffusion, as predicted for α-Li_3_PS_4_ both here (see below) and in the literature,^30,57^ very long MD trajectories are essential in achieving convergence of the Li displacement.^58^

A multitude of structural and chemical mechanisms have been proposed to explain the different Li-ion transport properties of γ-, β-, and α-Li_3_PS_4_. Perhaps the simplest of these mechanisms is the volume increase observed for the polymorph transitions from γ to β and then α. From our simulations, the γ to β polymorph transition results in a volume expansion of 6.70% (identical with the previous study by Zhang et al.),^45^ while the β to α polymorph transition produces an expansion of 5.18%. In the case of the γ to β polymorph transition, this volume increase has been associated with the change in the arrangement of PS_4_ tetrahedra at larger cell volumes.^25,45^ The polymorph transition from β- to α-Li_3_PS_4_ results in a more cubic-like symmetry and an almost random distribution of lithium sites, thereby leading to increased Li-ion disorder and subsequent conductivity. These increases in volume (and concomitant decreases in density) increase free transport volume in Li_3_PS_4_ and can induce the correlated ion transport events discussed herein.

As with other sulfide solid electrolytes, the anion (sulfur) sublattice is another critical feature that can influence the Li-ion conductivity of Li_3_PS_4_. The pioneering work of Wang et al.^12^ revealed a fundamental relationship between anion packing and corresponding Li-ion transport, whereby an underlying BCC anion framework that allows for direct Li hops between adjacent tetrahedral sites is most desirable for achieving high ionic conductivity. Wang et al.^12^ categorized the β-Li_3_PS_4_ anion framework as being more BCC than HCP. In contrast, Kim et al.^30^ used a polyhedral template matching method and found that the anion sublattices of both γ- and β-Li_3_PS_4_ are completely dominated by HCP frameworks, whereas α-Li_3_PS_4_ adopts a predominantly BCC anion framework with prominent tetrahedral–tetrahedral pathways. This finding was used to explain the predicted high Li-ion conductivity and low activation energy from AIMD simulations for α-Li_3_PS_4_.^30^

Li–Li separation is also considered to be an important indicator and influencer of fast Li-ion transport in Li_3_PS_4_^57^ (and indeed other Li-based solid electrolytes).^37,59,60^ For example, Kaup et al.^57^ suggested that some of the very short Li–Li distances in α-Li_3_PS_4_ may be responsible for the high Li-ion diffusion observed with AIMD.^30^ It was proposed that these short distances may result in concerted ion migration due to the increased repulsion between Li ions, as previously reported in Li_3.25_Si_0.25_P_0.75_S_4_.^61^ Nevertheless, the authors also noted that similarly short Li–Li distances are also present in β-Li_3_PS_4_ and therefore cannot be entirely responsible for the fast Li-ion transport in α-Li_3_PS_4_ compared to β-Li_3_PS_4_.^57^

Anion libration/rotation and associated “paddlewheel” effects, as discussed above, represent additional mechanisms through which Li-ion transport can be enhanced in fast-ion conductors. In the case of Li_3_PS_4_, it is the movement of the tetrahedral PS_4_ groups that could have a significant effect on the Li-ion transport properties. For γ-Li_3_PS_4_, the ordered PS_4_ groups are aligned along the *c*-axis, which has been proposed to restrict their reorientation and suggested as one of the main underlying factors for its comparatively low Li-ion conductivity.^25,45^ Alternatively, in β-Li_3_PS_4_, the PS_4_ tetrahedra follow a zigzag arrangement,^25^ which has been hypothesized to give rise to notable PS_4_ reorientation and increased Li-ion conductivity.^45^ The increase in volume as a result of the γ to β polymorph transition (see above) has also been linked to the onset of possible PS_4_ reorientation in Li_3_PS_4_.^41,45^

It is currently unknown whether PS_4_ reorientation occurs in α-Li_3_PS_4_ and, if so, what effect, if any, it has on Li-ion conductivity in this polymorph. In the only existing computational study of α-Li_3_PS_4_, PS_4_ reorientation was only briefly considered, and it was stated to show only “limited displacement”.^30^ However, the tMSD for sulfur presented in the work of Kim et al.^48^ shows significant sulfur displacement indicative of substantial PS_4_ reorientation. Given the established relationship between volume, PS_4_ disorder, and PS_4_ reorientation, it would be very surprising if the PS_4_ groups in α-Li_3_PS_4_ did not play an important role in determining its Li-ion transport properties. To the best of our knowledge, this study is the first time that the role of PS_4_ in α-Li_3_PS_4_ has been explicitly considered.

**Figure 2:**
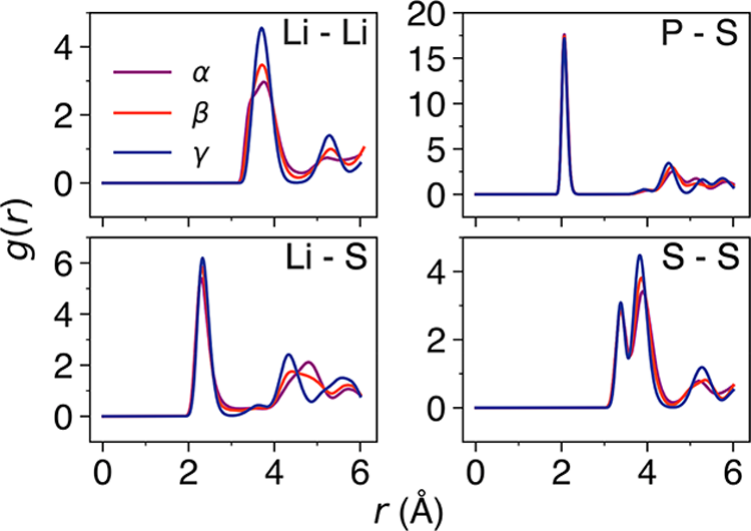
Li–Li, Li–S, P–S, and S–S RDFs for γ-, β-, and α-Li_3_PS_4_ at 400 K.

To relate the local structural factors of γ-, β-, and α-Li_3_PS_4_ to their Li-ion diffusion characteristics, we analyze the radial distribution functions (RDFs) for Li–Li, Li–S, P–S, and S–S in the three polymorphs. The RDFs at 400 K are displayed in Figure 2. For the Li–Li RDFs, there is a clear increase in peak broadening from γ- to β-Li_3_PS_4_ and then to α-Li_3_PS_4_, indicative of an increase in disorder. This supports the highest Li-ion diffusion coefficients and lowest activation energy found for α-Li_3_PS_4_ but does not explain the very similar Li-ion diffusivities calculated for γ- and β-Li_3_PS_4_ in Figure 1. This is perhaps indicative of a difference between the Li-ion diffusion mechanisms for γ- and β-Li_3_PS_4_ compared to α-Li_3_PS_4_. While the main peaks in the Li–S RDFs at ∼2.3 Å are similar, there is significant differences in the peaks at >4.0 Å for both β- and α-Li_3_PS_4_ compared to γ-Li_3_PS_4_. Similarly, subtle differences between the P–S and S–S RDFs of β- and α-Li_3_PS_4_ are also observable as a result of the distinct structural features of the three polymorphs. The RDFs presented here are in excellent agreement with those calculated with DFT^29,41^ and pair distribution functions from experiment.^26,62^ Equivalent RDFs at 1000 K are given in Figure S5.

**Figure 3:**
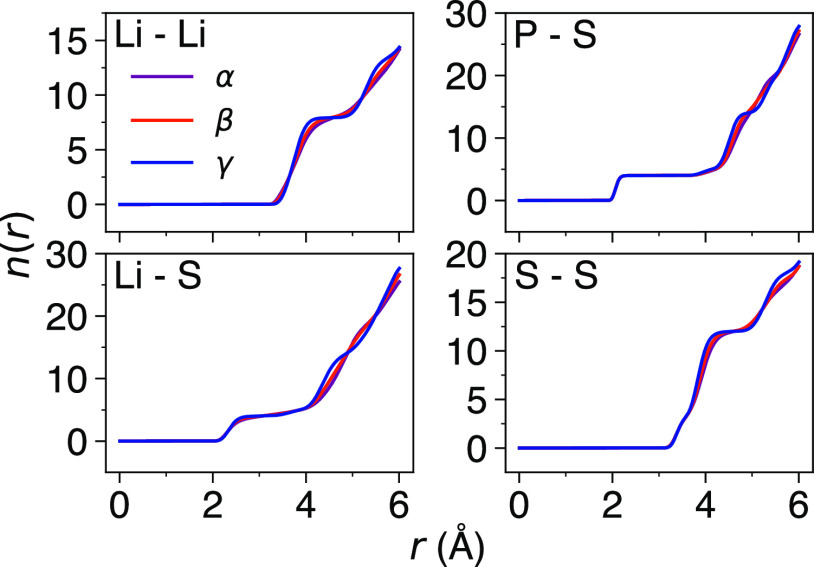
Integrated Li–Li, Li–S, P–S, and S–S RDFs for γ-, β-, and α-Li_3_PS_4_ at 400 K.

Analysis of the integrated RDFs provides useful information about the local coordination of a species. The integration of the Li–Li, Li–S, P–S, and S–S RDFs for γ-, β-, and α-Li_3_PS_4_ at 400 K is given in Figure 3. While the integrated RDFs are generally congruent, the increased disorder in β- and α-Li_3_PS_4_ compared to γ-Li_3_PS_4_ is once again clear. For example, in the case of Li–Li, the curves for β- and α-Li_3_PS_4_ are substantially smoother than the curve for γ-Li_3_PS_4_, which has a plateau at ∼4–5 Å. Similar behavior is also seen in the integrated P–S and S–S RDFs. The integrated RDFs for 1000 K are presented in Figure S6.

### 3.2 Influence of Li–Li Separation on Li-Ion Diffusion in Li_3_PS_4_

Given the important role that it is known to play in Li-ion conductors, including Li_3_PS_4_,^37,57,59,60^ here we explore the influence Li–Li interatomic distance and interaction have on Li-ion diffusion by explicitly removing the short-range Li–Li potential from the Li_3_PS_4_ model (see Methodology). The omission of this interatomic potential effectively removes the energetic benefit from Li ions maintaining their equilibrium Li–Li distance (3.4 Å), thereby allowing them to exhibit a broader range of interatomic separations. It is noteworthy that this is achieved without significantly altering the rest of the structure, as confirmed by the similarity between calculated lattice parameters both with and without the Li–Li potential (Table S2). The repulsive Coulombic interaction between Li ions is unaffected. It is noteworthy that such flexibility in tuning the interatomic potential model is only possible for analytical potentials. Achieving a similar result for potentials derived from machine learning would not have been possible (or would have required a complicated scheme to modify the training data or fitting method accordingly).

**Figure 4:**
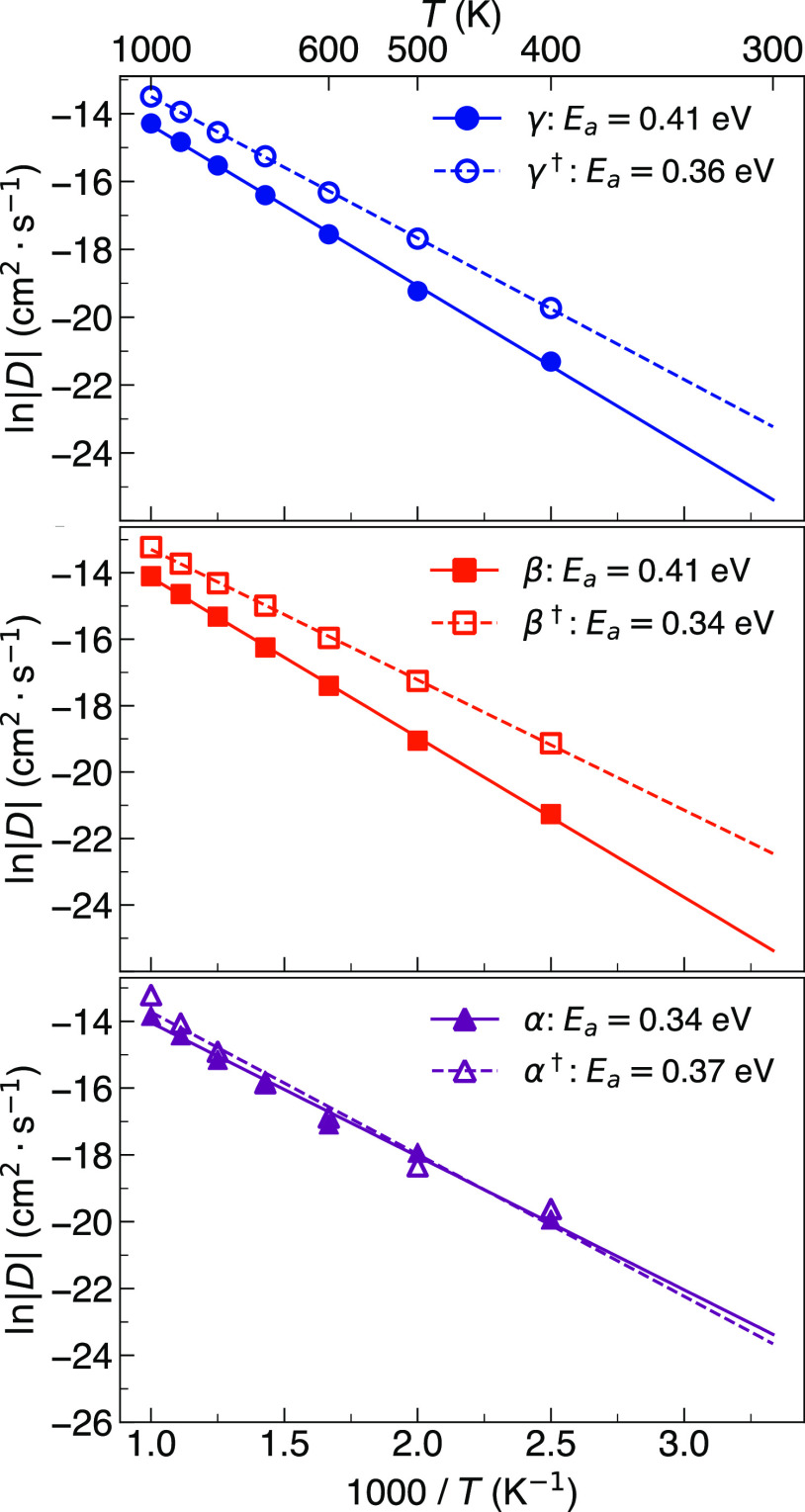
Arrhenius dependence of Li-ion diffusion in γ-, β-, and α-Li_3_PS_4_ with and without (†) short-range Li–Li potential included.

Arrhenius plots of the calculated Li-ion diffusion in γ-, β-, and α-Li_3_PS_4_ with the exclusion of the short-range Li–Li interatomic potential are presented in Figures 4 and S7. The inclusion of the Li–Li interatomic potential ultimately enforces an energetic penalty on Li ions becoming too close or too far apart. Under this condition, when a single Li ion hops, it can be assumed that it will drag/push the surrounding ions with it, thereby effectively enforcing concerted diffusion and discouraging discrete jumps. In the context of our results, given that discrete hops are expected in γ- and β-Li_3_PS_4_, when we remove the short-range Li–Li interatomic potential, the overall Li-ion diffusion should increase in these polymorphs because the Li ions can now hop independently. This is exactly the behavior we observe for γ- and β-Li_3_PS_4_ (Figures S4 and 4), which demonstrate significantly enhanced Li-ion diffusion and reduced activation energies compared to when the Li–Li potential is included.

In contrast, strikingly congruent plots of the high-temperature α polymorph can be observed in Figure 4 when comparing the Li-ion diffusion with and without the short-range Li–Li potential included. This suggests that, unlike for γ- and β-Li_3_PS_4_, concerted Li-ion diffusion occurs in this polymorph regardless of the inclusion of the Li–Li potential and for a wide range of Li–Li distributions. It may also be that the increased volume of α-Li_3_PS_4_ means that Li ions do not necessarily need to be in close contact for fast Li-ion diffusion in this polymorph. This would agree with the hypothesis of Kaup et al.^57^ in that the energy landscape in α-Li_3_PS_4_ is more important in determining its fast Li-ion conduction than the Li–Li distances. Furthermore, other factors may dominate the Li-ion diffusion mechanism in this polymorph, such as PS_4_ dynamics (see next section) and/or the role of the anion sublattice.^12,30,48^

**Figure 5:**
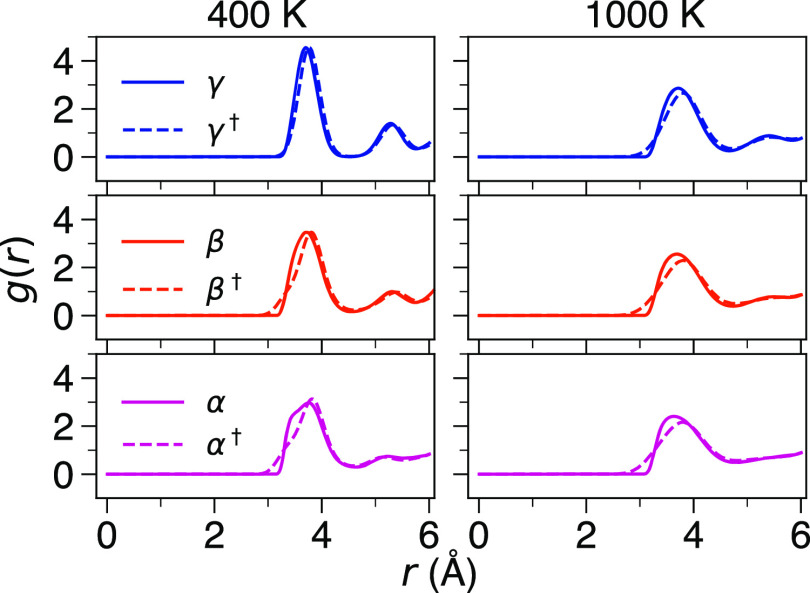
Comparison of Li–Li RDFs with and without (†) the short-range Li–Li interaction included at 400 and 1000 K.

To further verify these findings and understand the fundamental differences between the three polymorphs, we plot their respective Li–Li RDFs with and without the short-range Li–Li potential at 400 and 1000 K, as displayed in Figure 5. At 400 K, it is actually α-Li_3_PS_4_ that shows the greatest deviation between the two curves, with a sharper peak and smaller Li–Li distances observed for the case with short-range Li–Li potential excluded. Even though this polymorph experiences the greatest change in the Li–Li distribution, its Li-ion diffusion does not significantly change, as discussed above. This confirms that the influence of Li–Li distribution/separation in α-Li_3_PS_4_ does not significantly impact Li-ion diffusion and again that other ion transport mechanisms may dominate or that Li–Li correlation is enabled for a wide range of Li–Li distributions.

Similar changes in the Li–Li RDF profiles for γ-Li_3_PS_4_ at 1000 K and β-Li_3_PS_4_ at 400 and 1000 K are also observed but to a lesser extent. On the basis that the Li–Li distance strongly influences Li-ion diffusion in Li_3_PS_4_, as discussed above, these results indicate that, in contrast to α-Li_3_PS_4_, small changes in the Li–Li distributions in γ- and β-Li_3_PS_4_ have a dramatic influence on the magnitude of Li-ion diffusion. It is also noteworthy that, unlike for β- and α-Li_3_PS_4_, the Li–Li RDF for γ-Li_3_PS_4_ at 400 K with the short-range Li–Li potential removed does not show smaller Li–Li distances than the equivalent RDF with this potential present. This suggests the existence of an inhibiting structural factor in this polymorph perhaps related to its increased density, order, and/or PS_4_ dynamics (see the next section), which may well also be associated with its relatively low Li-ion diffusion. At 1000 K, more prominent and relatively similar changes in the RDFs occur across each polymorph to more disordered Li–Li distributions. For completeness, the integration of these RDFs is presented in Figure S8.

To further understand this dependence and highlight the temporal and structural dependence of the ion transport mechanisms, we calculate and plot the self-part of the van Hove correlation function (*G*_s_) for the Li–Li interaction in Li_3_PS_4_, as follows:
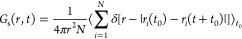
2where *G*_s_(*r*,*t*) is a function of the Li–Li pair distance *r* at simulation time *t*. The angular brackets denote the ensemble average from an initial time *t*_0_, *N* is the number of Li ions in the system, δ[ ] is the one-dimensional Dirac delta function, and *r*_*i*_(*t*) denotes the position of the *i*th Li ion at time *t*. For a given *r* and *t*, *G*_s_(*r*,*t*) or its transformed form, *r*^2^*G*_s_(*r*,*t*), provides the probability distribution of distances an atom *i* has reached from its starting position in a time *t*.

**Figure 6:**
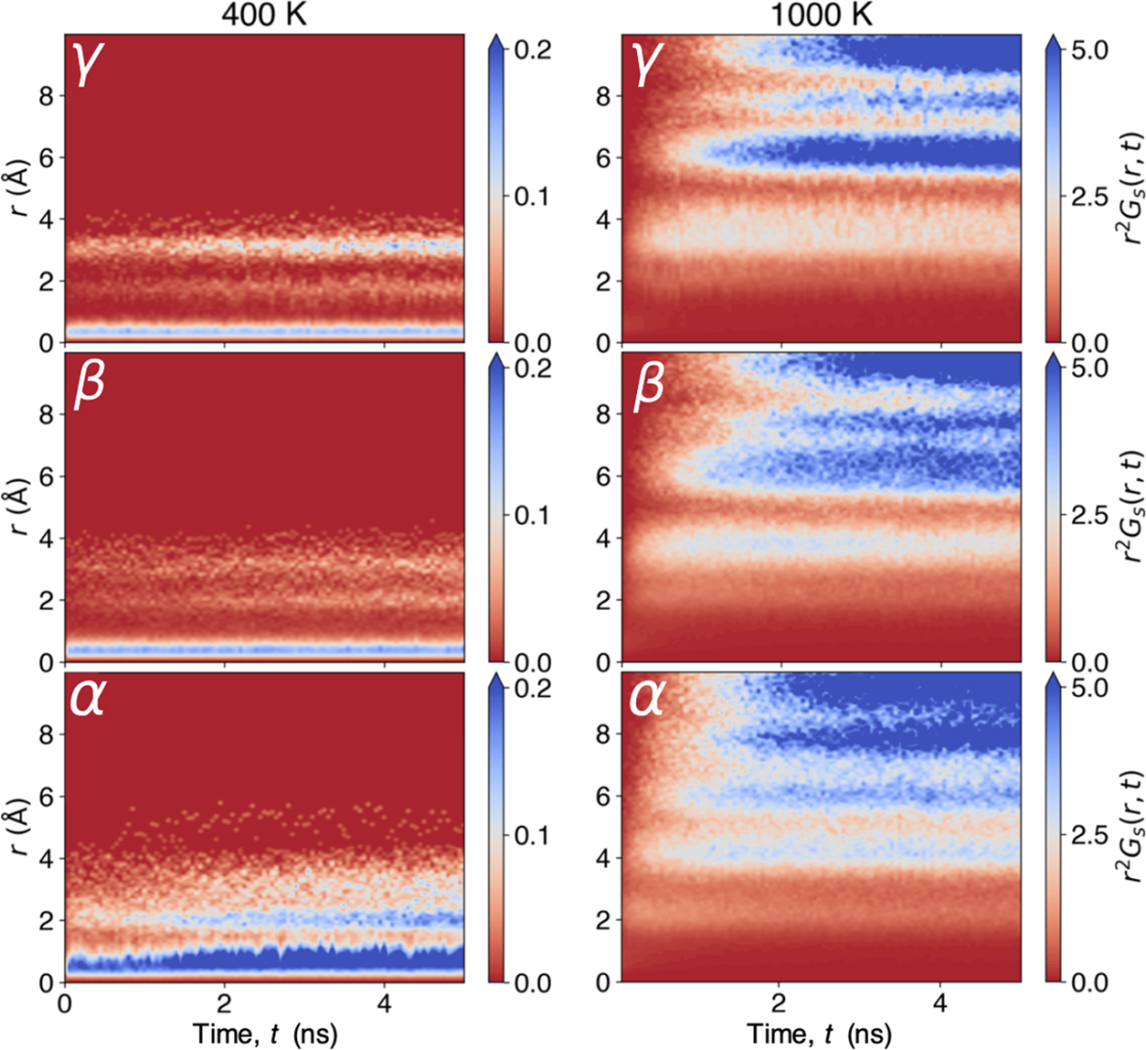
Transformed version of the self-part of the van Hove correlation function for γ-, β-, and α-Li_3_PS_4_ at 400 and 1000 K.

In Figure 6, *r*^2^*G*_s_(*r*,*t*) is plotted as a function of *r* at 400 and 1000 K for all three polymorphs. At 400 K, all three polymorphs demonstrate a peak between 0.0 and 0.5 Å, which can be ascribed to typical atomic equilibrium vibrations. This probability shows negligible time dependence in the γ and β polymorphs. Conversely, in the α polymorph, we observe that this low-lying peak broadens over time, which highlights an important difference between the structure of the α polymorph and the other polymorphs; the increased volume and open structure of the α polymorph causes the Li sublattice to be very disordered, with many partially occupied Li sites lying very close (within 1.5 Å) to one another. In fact, this peak becomes broad enough that it begins to overlap into the region of ∼1.5 Å, indicating that Li ions are not simply engaging in very high amplitude local oscillations, but are also moving to occupy the nearest-neighbor sites. The availability of multiple sites within proximity to one another enables facile Li-ion diffusion, as Li ions can make many short jumps that accumulate into long-range transport. This is supported by the introduction of another high-intensity and time-dependent peak at ∼2 Å, indicating that Li ions are mobile enough to migrate far from their initial position, even at temperatures as low as 400 K.

At 1000 K, the Li ions in all polymorphs now have enough energy to easily hop between sites, and we observe new peaks at larger displacements, indicating that Li-ion transport has reached the long-range diffusion regime. Despite all polymorphs demonstrating this apparent increase in diffusion, this is clearly most prominent in the α polymorph, closely followed by the β and γ polymorphs. It is important to note that although not visible at the scale used for the 1000 K plots, the local oscillatory peaks (i.e., 0–0.5 Å) are initially present for the γ and β polymorphs but fade quickly as ions diffuse away from their initial positions. For the α polymorph, ions diffuse so quickly that the peak is not visible (Figure S9).

### 3.3 Influence of PS_4_ Librations on Li-Ion Diffusion in Li_3_PS_4_

**Figure 7:**
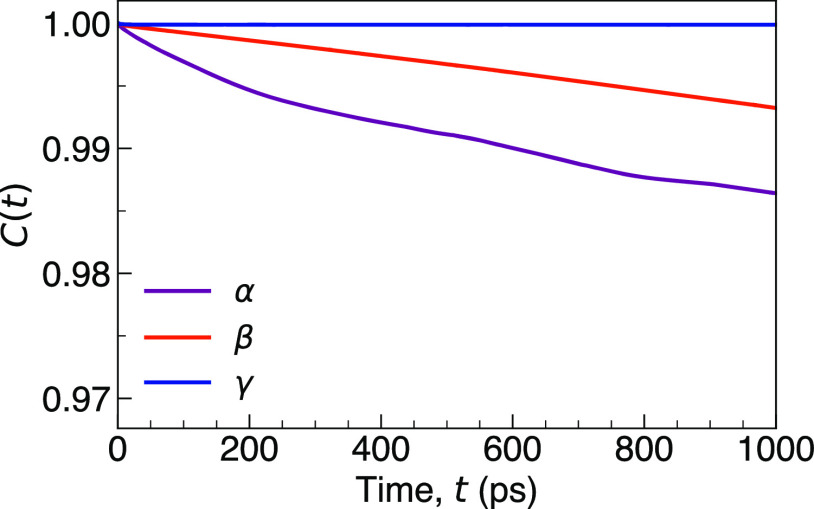
Vector autocorrelation function of PS_4_ anions in γ-, β-, and α-Li_3_PS_4_ at 1000 K. *C*(*t*) = ⟨**u**(*t* + *t*_0_)·**u**(*t*_0_)⟩, where **u** is a unit vector defined from the anion center of mass (P atom) to covalently bonded S atoms.

As discussed above, it has been proposed that the rotation/libration of translationally static anion groups can result in more facile Li-ion diffusion in solid electrolytes and thus higher ionic conductivities.^41−45^ The tendency for ionic molecular crystals to exhibit such characteristics is dependent on their structure and the high polarizability of the static framework, which have both been proposed to be features of Li_3_PS_4_.^45^ The degree of PS_4_ motion is ascertained by calculating their vector reorientational autocorrelation function from the molecular dynamics trajectories (Figure 7). This function evaluates the probability of the PS_4_ orientation remaining self-correlated over time, and therefore a fast decrease in the function indicates rapid rotation of the PS_4_ anions. As shown in Figure 7, while the autocorrelation function for γ-Li_3_PS_4_ remains unchanged as a function of time, indicating a lack of PS_4_ reorientation, the functions for β- and α-Li_3_PS_4_ show clear decays with simulation time, thereby illustrating the presence of PS_4_ libration in these polymorphs. This analysis provides the first direct evidence of PS_4_ libration in α-Li_3_PS_4_ and furthermore suggests that it is more significant in this polymorph than in β-Li_3_PS_4_.

**Figure 8:**
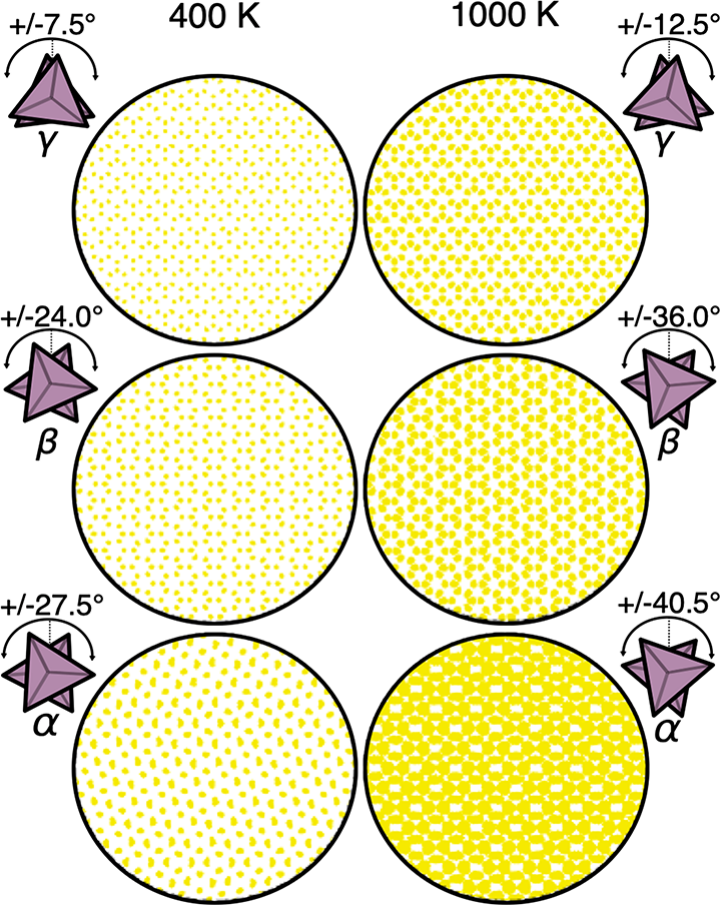
Density plots and maximum angular displacements of PS_4_ tetrahedra in γ-, β-, and α-Li_3_PS_4_ at 400 and 1000 K.

The extent of PS_4_ motion in Li_3_PS_4_ can be quantified and visualized by tracking the positions of the PS_4_ groups during the entirety of the simulations to calculate their maximum angular displacements and plotting their density profiles, respectively, as shown in Figure 8. It is clear that we only observe libration of the PS_4_ groups in all three polymorphs rather than complete/free rotation at both the lowest and highest temperatures considered. As expected, the level of PS_4_ libration is higher in β-Li_3_PS_4_ (maximum angular displacements of ±24° and ±36° at 400 and 1000 K, respectively) than in γ-Li_3_PS_4_ (maximum angular displacements of ±7.5° and ±12.5° at 400 and 1000 K, respectively). In agreement with the vector reorientational autocorrelation function analysis, Figure 8 also shows that α-Li_3_PS_4_ exhibits the highest level of PS_4_ librational disorder (maximum angular displacements of ±27.5° and ±40.5° at 400 and 1000 K, respectively). Although the calculated maximum angular displacements are small, it was reported by Smith and Siegel^41^ that even relatively small librations can be closely linked to Li-ion hopping.

To isolate the effect of PS_4_ librational motion on Li-ion diffusion in Li_3_PS_4_, we inhibit the movement of these polyanions to varying degrees and then recalculate the Li-ion diffusion in the three polymorphs. By applying a spring force independently to each ion in the PS_4_ groups, we were able to tether the PS_4_ groups to their initial positions by varying magnitudes (i.e., 1, 5, and 10 eV Å^–2^). The use of this tethering function, rather than the stringent fixing or “freezing” often observed in DFT studies,^41,63−65^ provides additional flexibility in decoupling the structural and dynamic energy landscapes in solid electrolytes. This is because the tethering still permits local thermal vibrations of the ions, whereas completely fixing the ions prevents these librations altogether, resulting in a cagelike structure and the inability for sublattice constituents to shift to allow for migrating mobile ions. The restrictive effect of applying a spring force to the PS_4_ polyanions is clearly illustrated by comparing their density plots in Figure S10 for a spring constant of 10 eV Å^–2^ with those in Figure 8 with no spring constant.

**Figure 9:**
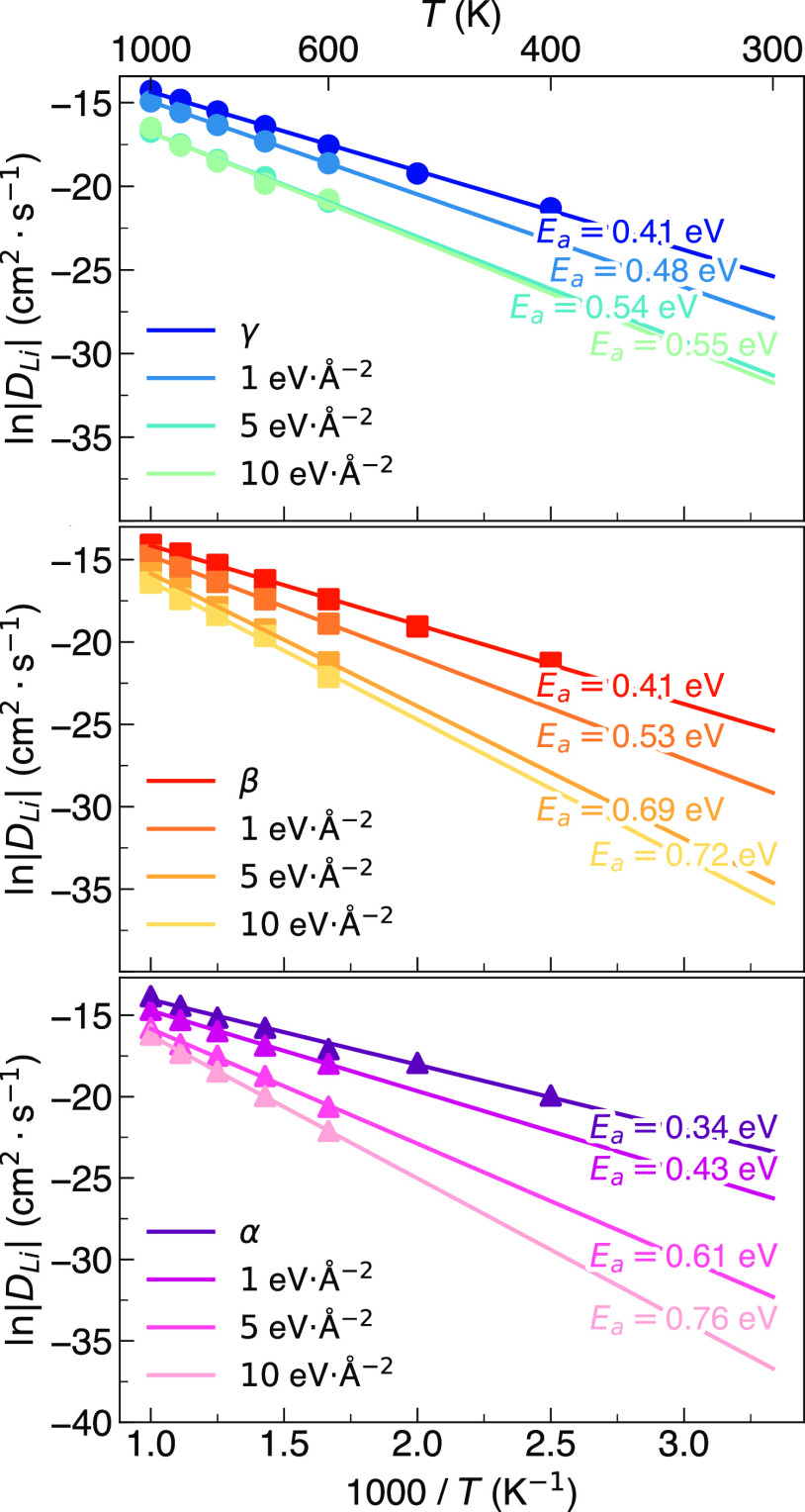
Arrhenius dependence of Li-ion diffusion in γ-, β-, and α-Li_3_PS_4_ as a function of spring force applied (i.e., 0, 1, 5, and 10 eV Å^–2^) to tether PS_4_ groups.

Arrhenius plots of the calculated Li-ion diffusion in γ-, β-, and α-Li_3_PS_4_ as a function of the spring constant applied are presented in Figure 9. It should be noted that the results at 400 and 500 K are not displayed because the Li-ion diffusion becomes too weak to be accurately modeled at the system and time scales adopted here. With the introduction of a spring force to the PS_4_ groups for all three polymorphs, we observe an immediate decrease in Li-ion diffusivity and an increase in activation energy with increasing spring constant from 1 to 10 eV Å^–2^. The effect of tethering the PS_4_ groups is weakest for γ-Li_3_PS_4_, which is to be expected given that this polymorph does not exhibit significant PS_4_ librational motion due to its high density and ordered nature (see Figures 8 and S9).^24−27^ For β- and α-Li_3_PS_4_, this effect is far more pronounced. While it has been well established that β-Li_3_PS_4_ exhibits significant PS_4_ libration,^45^ it is currently unclear to what extent it influences the Li-ion conductivity of α-Li_3_PS_4_.

**Figure 10:**
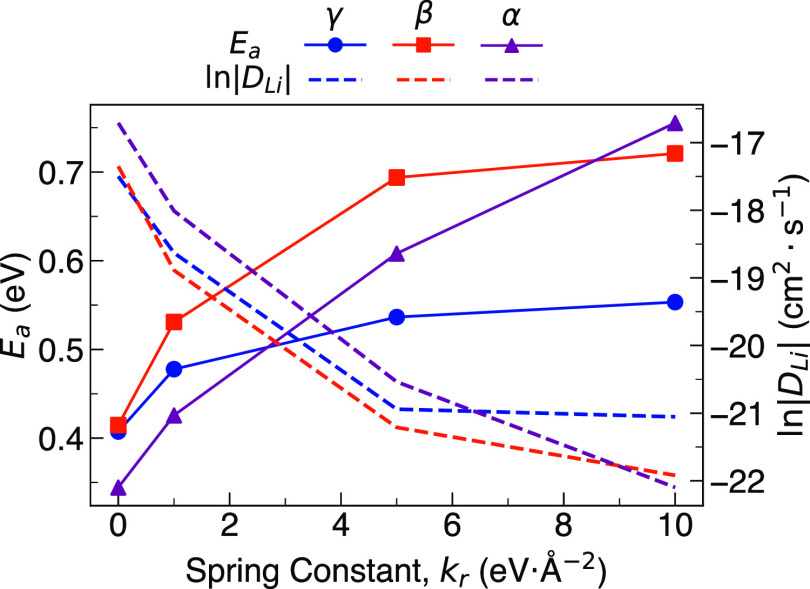
Comparison of activation energies and diffusivity (at 600 K) of γ-, β-, and α-Li_3_PS_4_ as a function of the applied spring force (i.e., 0, 1, 5, and 10 eV Å^–2^).

Figure 10 summarizes the calculated Li-ion diffusion coefficients at 600 K and activation energies for γ-, β-, and α-Li_3_PS_4_ as a function of the spring force applied to tether the PS_4_ groups. Although the effect of tethering the PS_4_ groups is weakest for γ-Li_3_PS_4_, it is still far from negligible and results in a significant reduction in Li-ion diffusion of several orders of magnitude and an increase in activation energy to 0.55 eV for a spring force of 10 eV Å^–2^. Nevertheless, it is also clear from Figure 10 that the effect of tethering the PS_4_ groups in this polymorph rapidly plateaus as a function of the spring constant. It is likely that this plateau represents the point at which the contribution of polyanionic motion to the Li-ion dynamics is limited entirely and where the Li-ion diffusivity is dependent on other structural and chemical characteristics.

In contrast to γ-Li_3_PS_4_, this plateau is only reached at larger spring constants of >5 eV Å^–2^ for β-Li_3_PS_4_, and for α-Li_3_PS_4_, it is still not achieved even at a spring constant of 10 eV Å^–2^. These results show that both the influence and magnitude of PS_4_ librational motion in β- and α-Li_3_PS_4_ are significantly larger than in γ-Li_3_PS_4_. Although this is perhaps to be expected given the proposed role of PS_4_ librational dynamics in β-Li_3_PS_4_, the dramatic decrease in Li-ion diffusion and increase in activation energy from 0.41 to 0.72 eV with a spring constant of 10 eV Å^–2^ are the first clear quantitative estimations of this effect. It is noteworthy that at a spring constant of 10 eV Å^–2^ α-Li_3_PS_4_ exhibits the lowest Li-ion diffusion and highest activation energy of all three polymorphs. This finding represents the first verification, computational or experimental, of the critical relationship between cation and anion dynamics in α-Li_3_PS_4_.

Furthermore, this unique analysis provides evidence that the increased volume of the α-Li_3_PS_4_ is not independently a major driver for its Li-ion diffusion. If the volume effect was the main driver of diffusion in this polymorph, then we would still expect it to exhibit the greatest Li-ion diffusion regardless of our manipulation of the PS_4_ groups. Nevertheless, the volume effect remains important in facilitating PS_4_ libration and therefore Li-ion transport. Our findings therefore strongly suggest that anion dynamics are a major source of the high ionic mobility in α-Li_3_PS_4_ as well as in β-Li_3_PS_4_. They also highlight an important consideration when considering the stabilization of α-Li_3_PS_4_ at room temperature, namely, to maintain high ionic conductivity, it is not enough to simply stabilize and expand the structure through, for example, doping, if this procedure also hinders the librational dynamics that give this polymorph its desirable properties.

## 4 Conclusions

A thorough understanding of the interplay between cation and anion dynamics is pivotal to the design and improvement of solid electrolyte materials for solid-state batteries. In this study, we utilize large-scale molecular dynamics to provide new insights into how and to what extent cation and anion dynamics influence Li-ion transport in the three primary polymorphs of the prominent thiophosphate solid electrolyte, Li_3_PS_4_. In this context, we differentiate the many interlinked facets encapsulated in the somewhat nebulous term “correlation” and elucidate their subsequent presence and dominance in each polymorph of Li_3_PS_4_.

**Figure 11:**
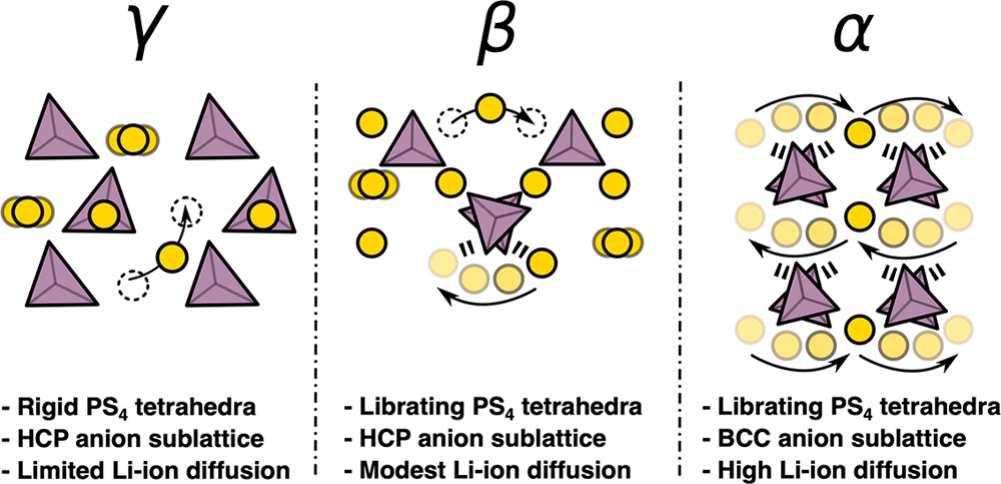
Schematic depicting the main structural and dynamic properties responsible for the different magnitudes of Li-ion transport in γ-, β-, and α-Li_3_PS_4_.

As summarized schematically in Figure 11, by disentangling the cation and anion dynamics in Li_3_PS_4_, we have been able to identify the main structural and chemical driving forces responsible for different levels of Li-ion diffusion in γ-, β-, and α-Li_3_PS_4_. While we have considered these distinctly, it is once again important to clarify that these qualities are not mutually exclusive, with distinction between association and causation decisively untrivial. Fast/superionic diffusion can therefore be considered as the incoherent dynamics of multiple mechanisms simultaneously.

The key findings of our study are highlighted as follows:

- The Li-ion diffusion coefficients and corresponding activation energies in γ-, β-, and α-Li_3_PS_4_ are calculated and related to the unique structural and chemical characteristics of each polymorph, including cell volume, anion sublattice, order/disorder, and local structures.
- The influence of Li–Li correlation and separation is revealed based on comparative simulations with and without the inclusion of a Li–Li interatomic potential. While the distance between Li ions is crucial in establishing the magnitude of Li-ion diffusion in γ- and β-Li_3_PS_4_, the α-Li_3_PS_4_ structure can adopt a wider range of Li–Li distances and still exhibit the same high level of Li-ion diffusion.
- The role of anion librational motion in enhancing the Li-ion dynamics in Li_3_PS_4_ is also isolated. We find that in β- and α-Li_3_PS_4_ the tethering of polyanions to restrict their motion has a more significant detrimental effect on Li-ion diffusion than in γ-Li_3_PS_4_, suggesting that polyanionic motion is an important source of the high diffusion in these polymorphs.

Enhancing our fundamental understanding of these factors and their influence on ion transport is crucial for the future optimization of new solid electrolytes, and it is highly anticipated that the analysis presented here will be extended to analogous systems for the development of fast-ion conductors. In addition to the structural motifs and dynamics of the ideal bulk Li_3_PS_4_ crystal lattices considered, the methods used herein are equally applicable to consider other important solid electrolyte properties, including doping, grain boundaries, and electrolyte–electrode interfaces.

## Supporting Information Available

Potential model for Li_3_PS_4_; calculated and experimental lattice parameters and atomic positions; crystal structures of Li_3_PS_4_; Arrhenius plots of Li-ion diffusion in stoichiometric Li_3_PS_4_; trajectories of Li ions in Li_3_PS_4_ at 400 K; radial distribution functions for Li_3_PS_4_ at 1000 K; Arrhenius plots of Li-ion diffusion in Li_3_PS_4_ without short-range Li–Li potential; integrated radial distribution functions for Li–Li at 400 and 1000 K; transformed version of self-part of van Hove correlation function for Li_3_PS_4_ at 1000 K; density plots of PS_4_ librational motion in Li_3_PS_4_ at 400 and 1000 K with a spring constant of 10 eV Å^–2^ applied (PDF)
