# Supplementary material for: Disentangling Cation and Anion Dynamics in Li3PS4 Solid Electrolytes
Source: Chem Mater. 2022 Nov 9;34(23):10561–71. doi: 10.1021/acs.chemmater.2c02637 (PMC9753599; doi:10.1021/acs.chemmater.2c02637)
Supplement: Supplementary file 1 — cm2c02637_si_001.pdf [file cm2c02637_si_001.pdf]

—Supporting Information—

Disentangling Cation and Anion Dynamics in Li_3PS_4 Solid Electrolytes

Frazer N. Forrester[1], James A. Quirk[1], Theodosios Famprikis[2] and James A. Dawson[1,3*]

[1]Chemistry – School of Natural and Environmental Sciences, Newcastle University, Newcastle upon Tyne, NE1 7RU, UK

[2]Department of Radiation Science and Technology, Faculty of Applied Sciences, Delft University of Technology, 2629JB Delft, The Netherlands

[3]Centre for Energy, Newcastle University, Newcastle upon Tyne, NE1 7RU, UK

*Corresponding author. Email: james.dawson@newcastle.ac.uk

Table S1. Interatomic Morse potential parameters for Li_3PS_4 taken from Kim *et al.*[1]

Interaction	D	α	r_0 (Å)
Li–Li	0.058000000	3.9870	3.40378
Li–S	0.040755682	1.3988	3.20378
P–S	0.410420000	2.3287	2.20028
S–S	0.024096411	1.3585	4.28352

The effective atomic charges of Li, P and S are +0.88, +1.28 and -0.98, respectively.

Table S2. Comparison of calculated (0 K) and experimental lattice parameters for γ-, β- and α-Li_3PS_4 with and without the Li–Li interatomic potential included.

Lattice parameters (Å)			
	This work (with Li–Li)	This work (without Li–Li)	Experiment[2]
γ-Li_3PS_4			
a	7.48	7.61	7.71
b	6.44	6.50	6.54
c	5.93	6.06	6.14
β-Li_3PS_4			
	This work (with Li–Li)	This work (without Li–Li)	Experiment[2]
a	12.81	12.83	12.82
b	7.84	7.93	8.22
c	6.07	6.05	6.12
α-Li_3PS_4			
	This work (with Li–Li)	This work (without Li–Li)	Experiment[3]
a	8.88	8.93	8.64
b	8.86	8.91	9.05
c	8.15	8.17	8.48

Table S3. Comparison of calculated (0 K) and experimental atomic positions (given in parentheses and reproduced from Homma *et al.*[2] for γ- and β-Li_3PS_4 and Kaup *et al.*[3] for α-Li_3PS_4) for γ-, β- and α-Li_3PS_4. Disordered Li sites in β- and α-Li_3PS_4 have been omitted.

Atomic positions			
γ-Li_3PS_4			
Li(1)	0.247 (0.250)	0.332 (0.331)	0.984 (0.018)
Li(2)	0.999 (0.000)	0.169 (0.145)	0.482 (0.486)
P	0.000 (0.000)	0.830 (0.822)	0.999 (0.994)
S(1)	0.227 (0.219)	0.678 (0.672)	0.880 (0.886)
S(2)	0.999 (0.000)	0.134 (0.108)	0.881 (0.888)
S(3)	0.999 (0.000)	0.826 (0.805)	0.337 (0.323)
β-Li_3PS_4			
P	0.090 (0.087)	0.272 (0.250)	0.130 (0.172)
S(1)	0.161 (0.155)	0.057 (0.046)	0.273 (0.285)
S(2)	0.931 (0.935)	0.279 (0.250)	0.197 (0.257)
S(3)	0.119 (0.107)	0.277 (0.250)	0.804 (0.837)
α-Li_3PS_4			
P	0.996 (0.000)	0.862 (0.831)	0.245 (0.250)
S(1)	0.314 (0.304)	0.484 (0.456)	0.256 (0.250)
S(2)	0.971 (0.000)	0.318 (0.295)	0.518 (0.554)

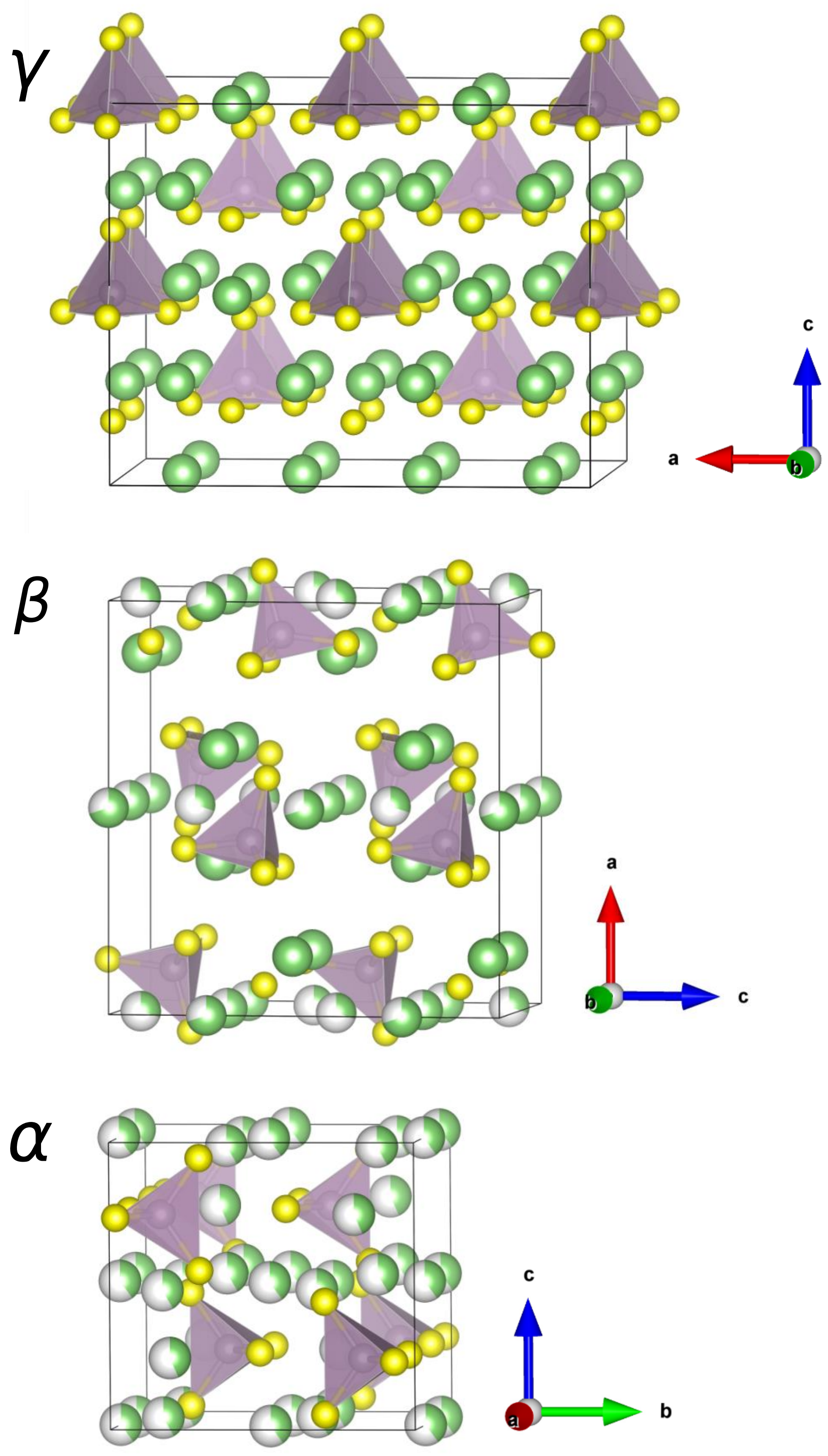

Figure S1. Crystal structures of γ-, β- and α-Li_3PS_4.

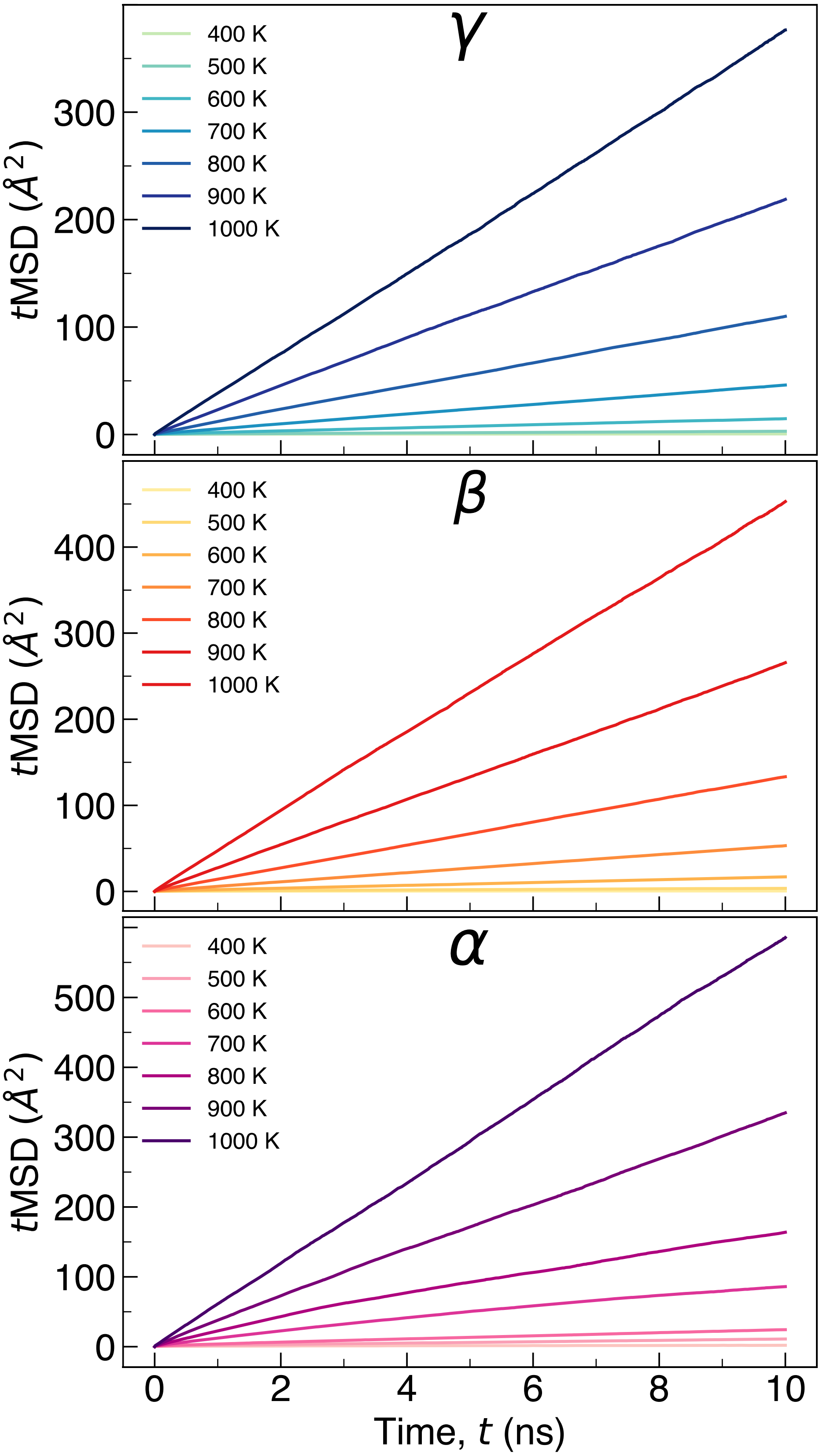

Figure S2. Example time-averaged mean square displacement (MSD) plots for γ-, β- and α-Li_3PS_4 at 1000 K.

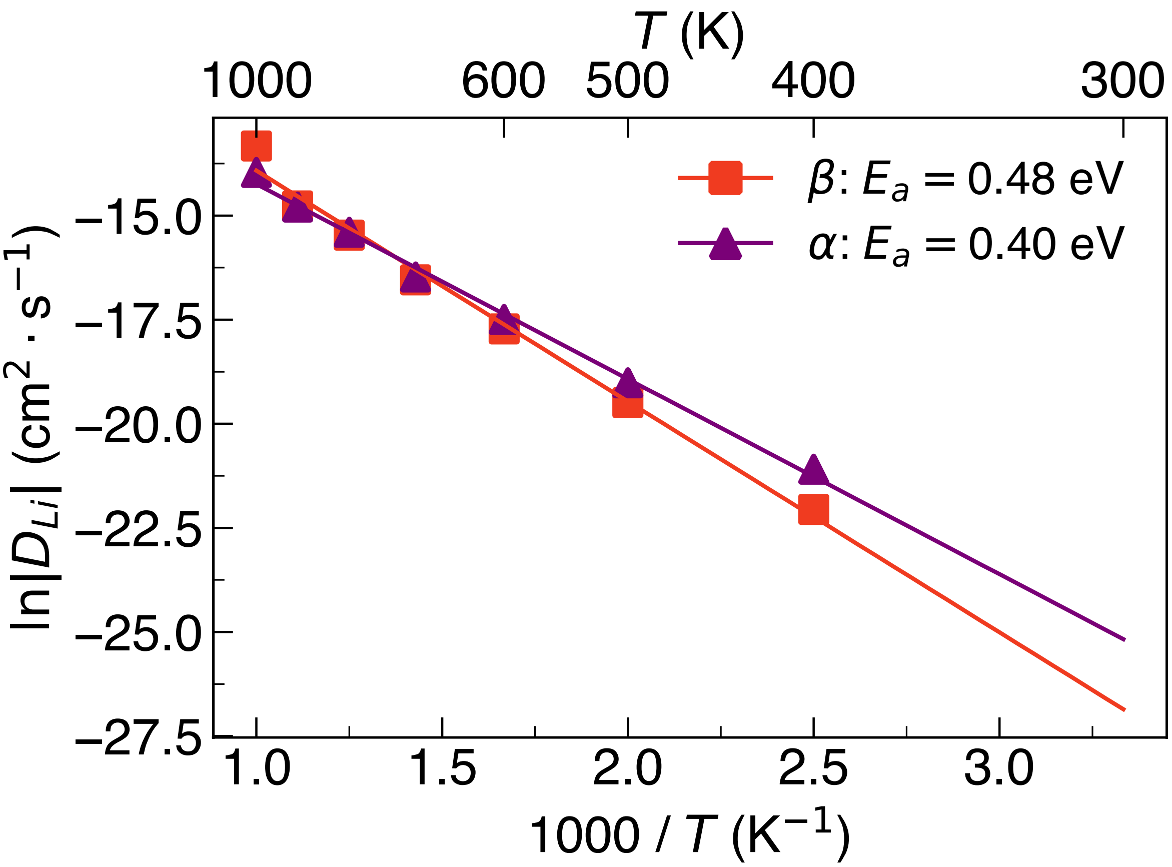

Figure S3. Arrhenius dependence of Li-ion diffusion in stoichiometric β- and α-Li_3PS_4. Stoichiometric γ-Li_3PS_4 did not exhibit Li-ion diffusion within the simulated temperature range of 400–1000 K.

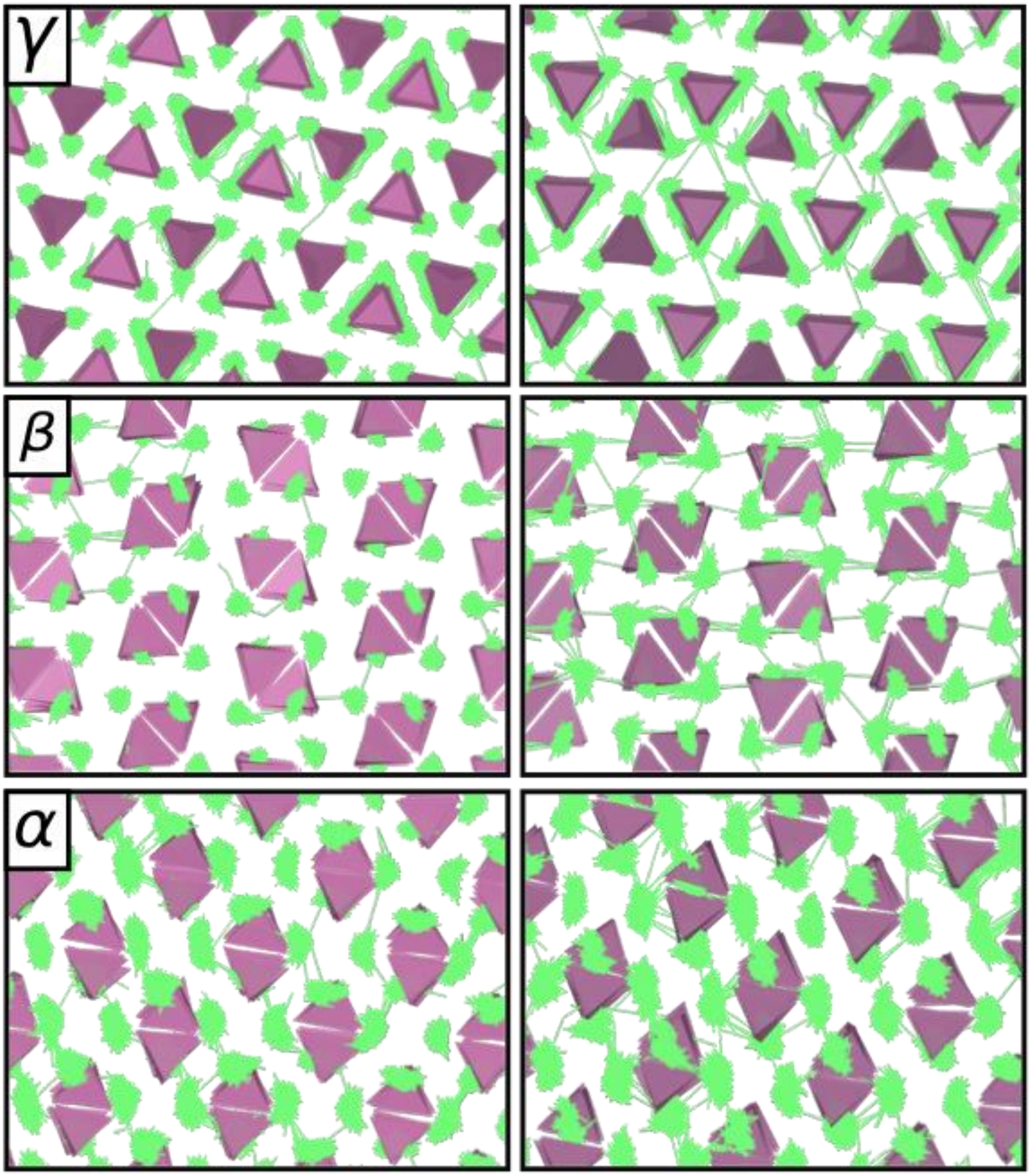

Figure S4. Real-space trajectories of Li ions throughout the 10 ns simulations with (left) and without (right) the short-range Li–Li potential included in γ-, β- and α-Li_3PS_4 at 400 K.

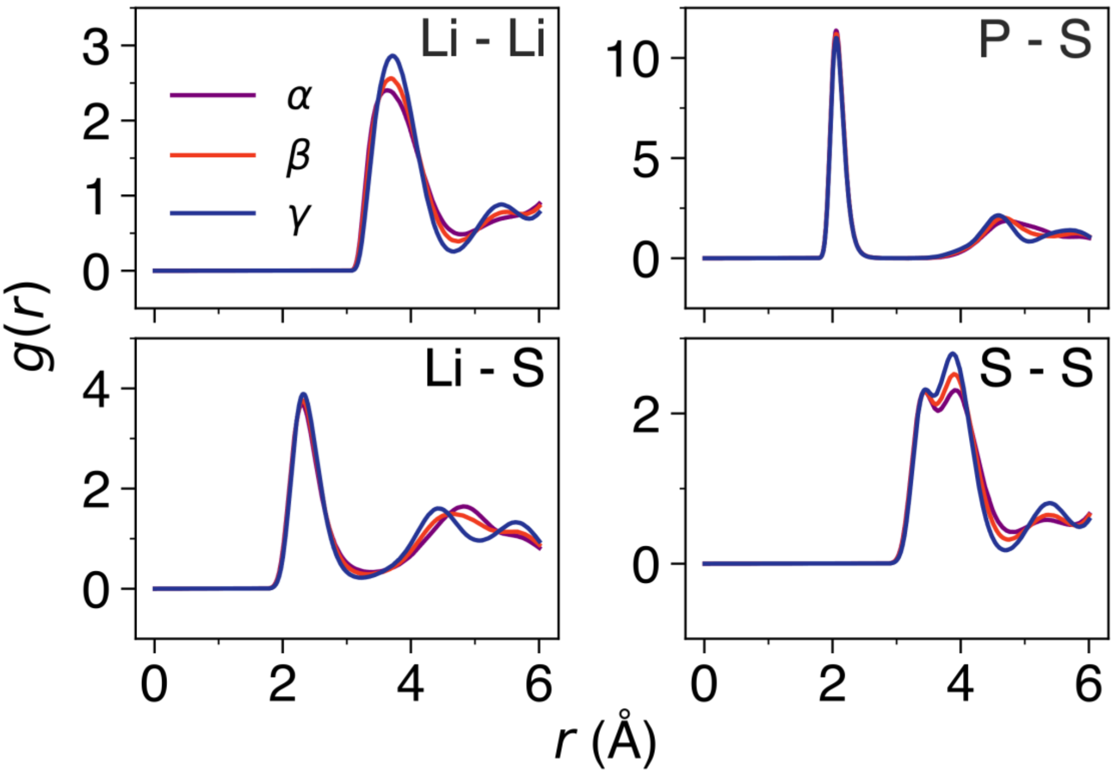

Figure S5. Radial distribution functions (RDFs) for γ-, β- and α-Li_3PS_4 at 1000 K.

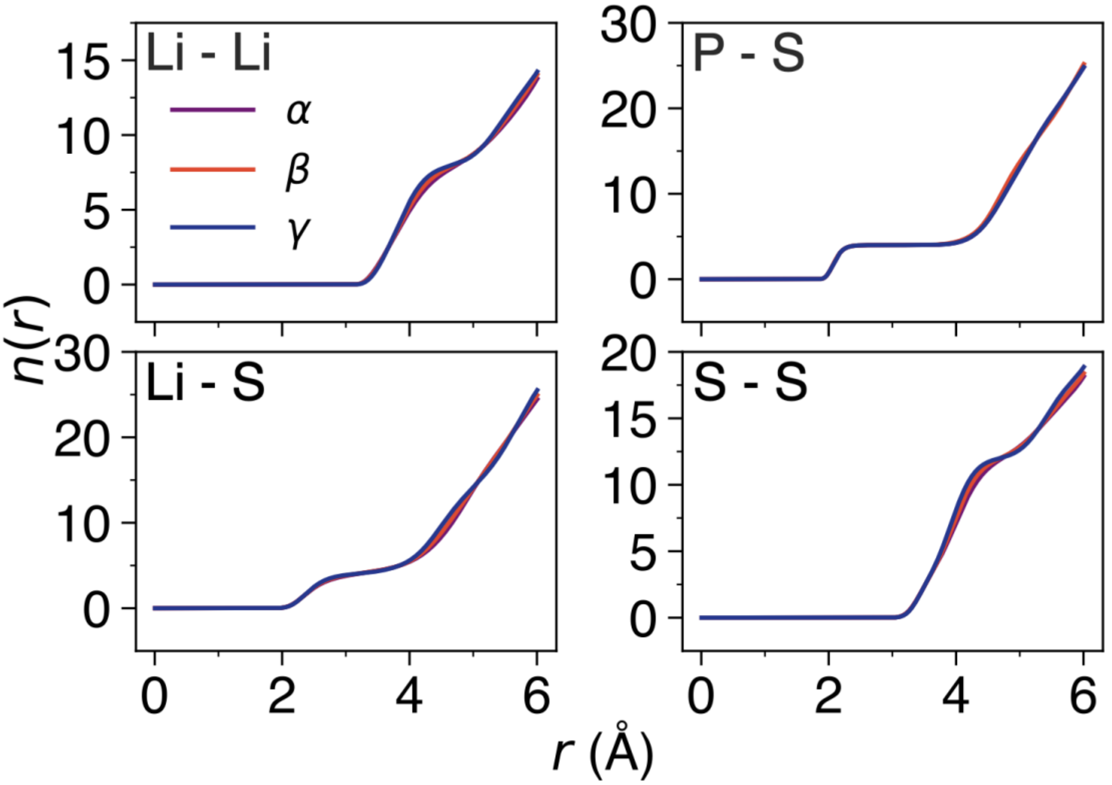

Figure S6. Integrated RDFs for γ-, β- and α-Li_3PS_4 at 1000 K.

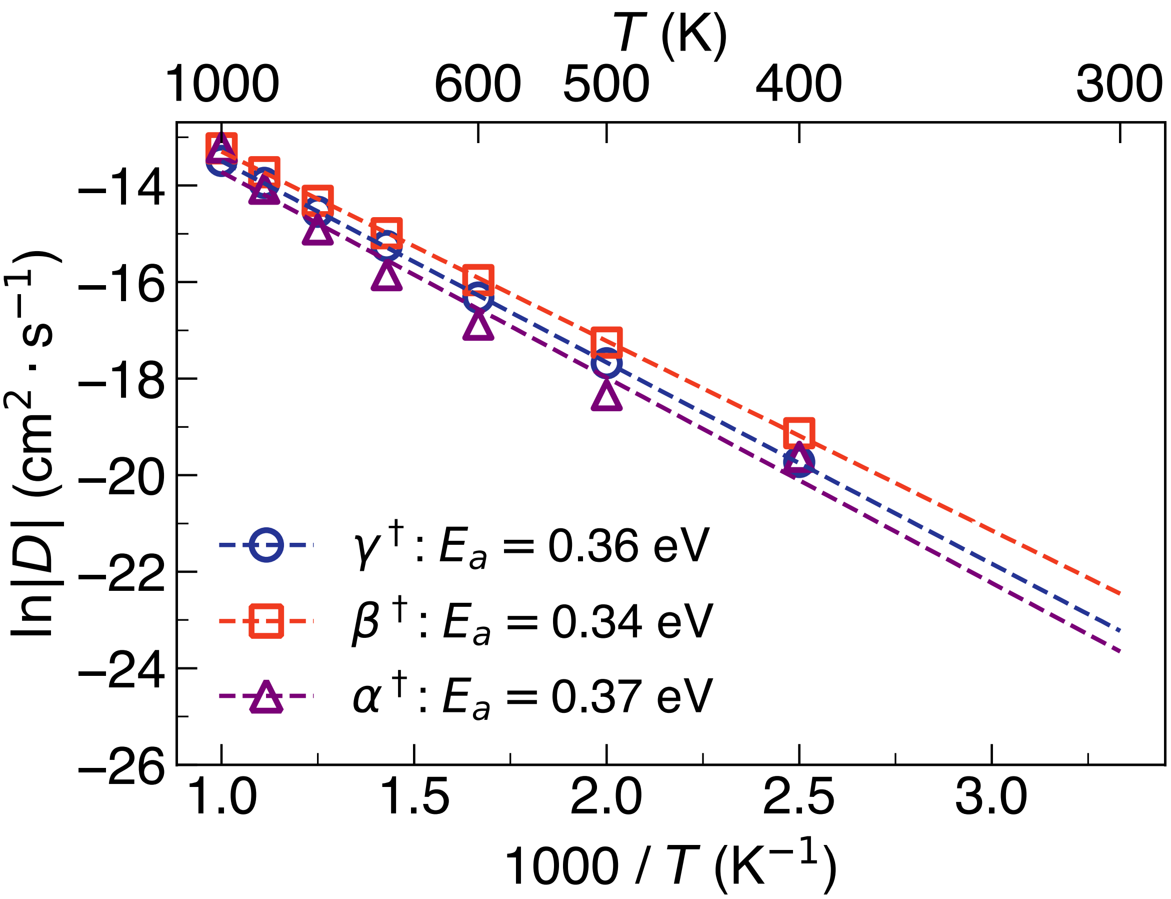

Figure S7. Arrhenius dependence of Li-ion diffusion in γ-, β- and α-Li_3PS_4 without short-range Li–Li potential included.

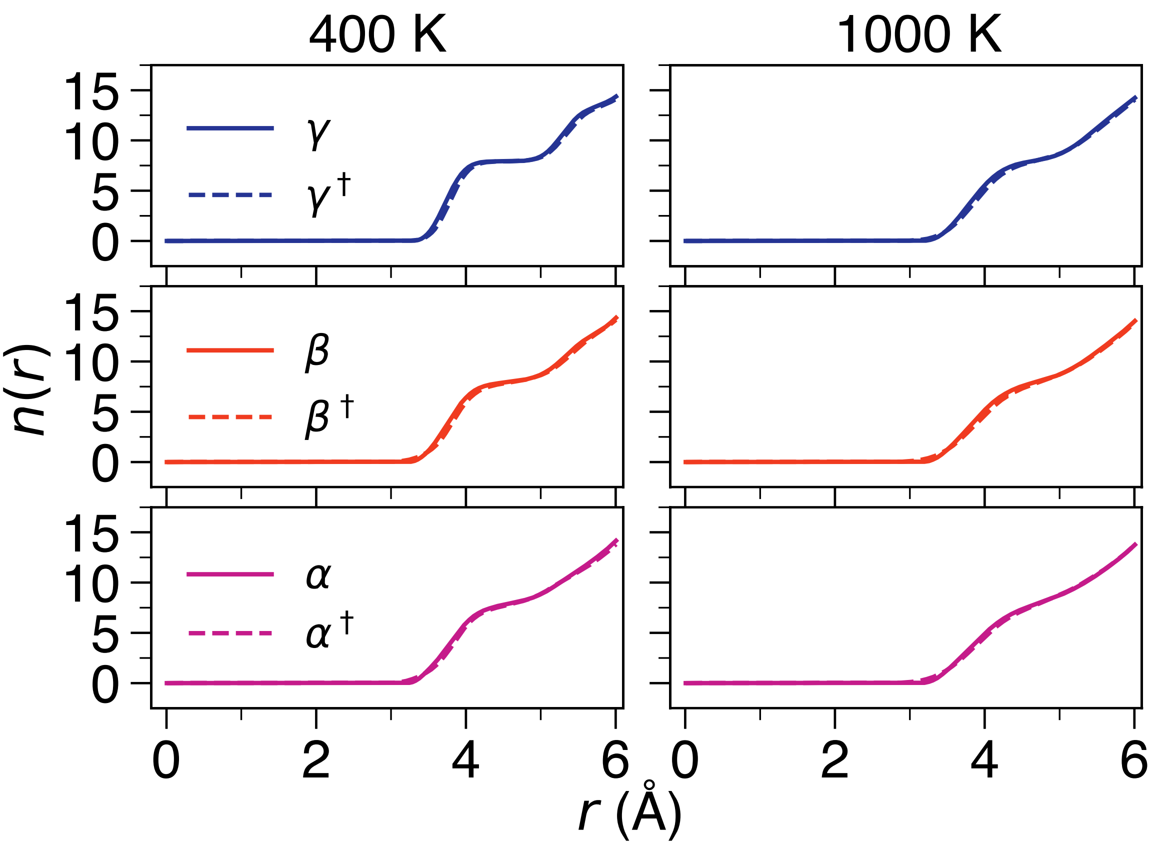

Figure S8. Comparison of integrated Li–Li RDFs for γ-, β- and α-Li_3PS_4 with and without (†) the short-range Li–Li interaction included at 400 and 1000 K.

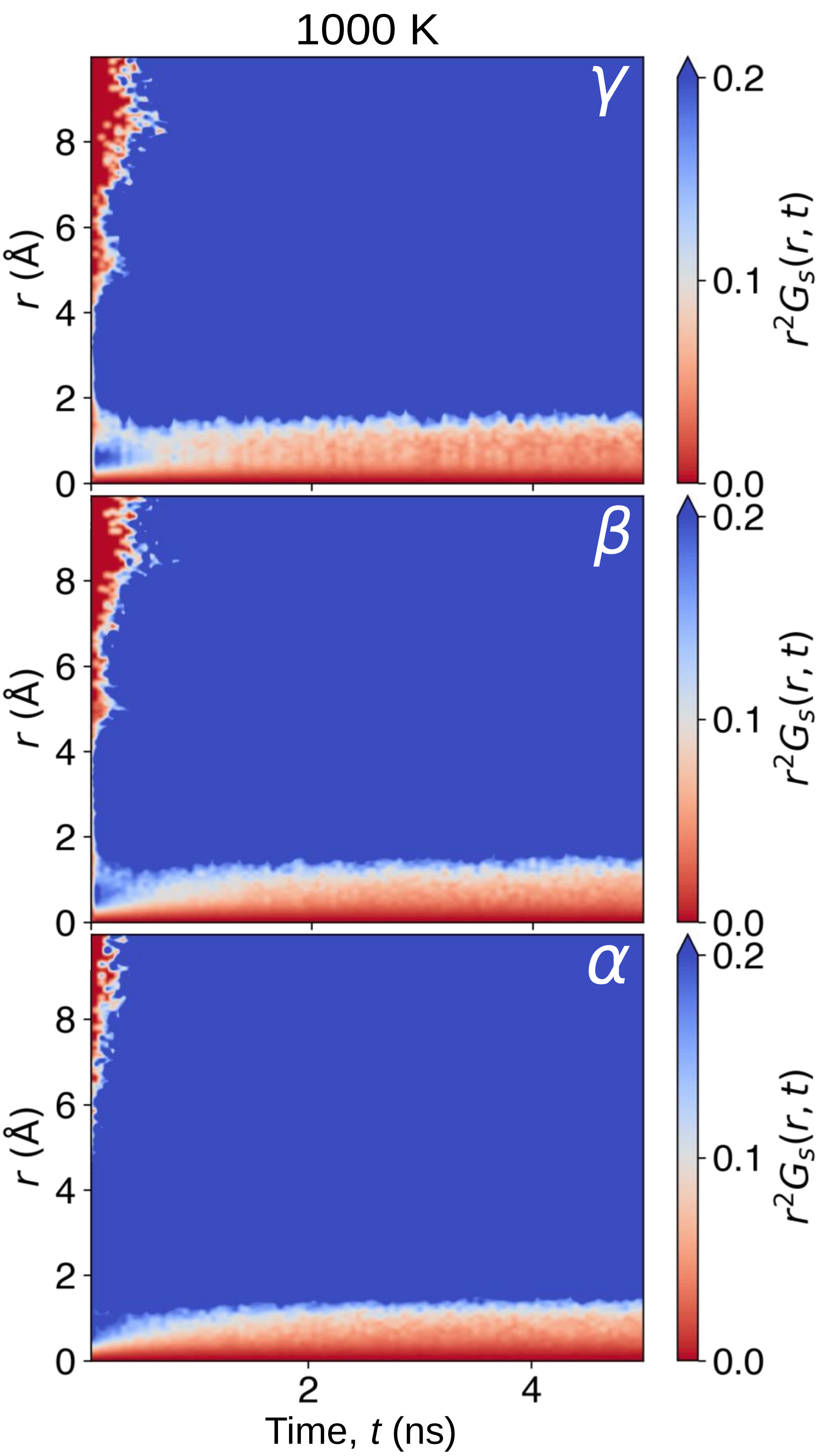

Figure S9. Transformed version of the self-part of the van Hove correlation function for γ-, β- and α-Li_3PS_4 at 1000 K. For γ- and β- Li_3PS_4, the peak at ~0.5 Å is visible for a short time but rapidly drops off as Li ions diffuse away from their initial sites. For α-Li_3PS_4, the peak disappears too rapidly to be visible.

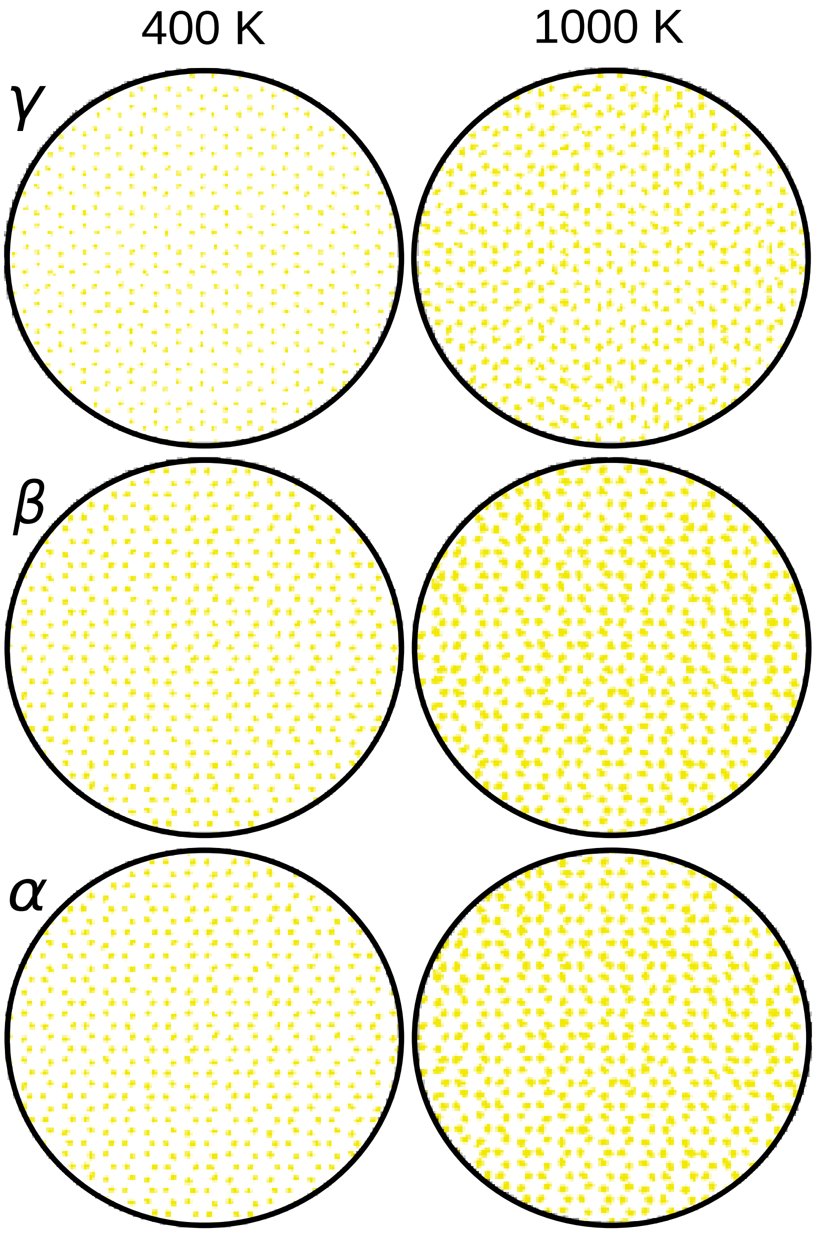

Figure S10. Density plots of PS_4 librational motion in γ-, β- and α-Li_3PS_4 at 400 and 1000 K with a spring constant of 10 eV $Å^{-2}$ applied.

REFERENCES

(1) Kim, J. S. *et al.* Atomistic Assessments of Lithium-Ion Conduction Behavior in Glass-Ceramic Lithium Thiophosphates. *ACS Appl. Mater. Interfaces* **11**, 13–18 (2019).

(2) Homma, K. *et al.* Crystal Structure and Phase Transitions of the Lithium Ionic Conductor Li_3PS_4. *Solid State Ionics* **182**, 53–58 (2011).

(3) Kaup, K.; Zhou, L.; Huq, A.; Nazar, L. F. Impact of the Li Substructure on the Diffusion Pathways in Alpha and Beta Li_3PS_4: An In Situ High Temperature Neutron Diffraction Study. *J. Mater. Chem. A* **8**, 12446–12456 (2020).
